# The Sex Difference in Gait Speed: How Do Sociodemographic, Lifestyle, Social, and Health Determinants Contribute?

**DOI:** 10.1093/geroni/igab046.642

**Published:** 2021-12-17

**Authors:** Lena Sialino, Laura Schaap, Sandra van Oostrom, Susan Picavet, Johannes Twisk, Monique Verschuren, Marjolein Visser, Hanneke Wijnhoven

**Affiliations:** 1 Vrije Universiteit Amsterdam, Amsterdam, Noord-Holland, Netherlands; 2 National Institute for Public Health and the Environment, Amsterdam, Noord-Holland, Netherlands; 3 National Institute for Public Health and the Environment, Bilthoven, Utrecht, Netherlands; 4 Amsterdam University Medical Centre, Amsterdam, Noord-Holland, Netherlands

## Abstract

This study explores whether a sex difference in sensitivity to (strength of the association) and/or in exposure to (prevalence) determinants of gait speed contributes to the observed lower gait speed among women compared to men. Data from the Longitudinal Aging Study Amsterdam (LASA) were used. In total 2407 men and women aged 55-81 years were included, with baseline measurements in 1992/2002 and follow-up measurements every 3-4 years for 15/25 years. Multivariable mixed model analysis was used to investigate sex differences in sensitivity (interaction term with sex) and in exposure to (change of the sex difference when adjusted) for socio-demographic, lifestyle, social and health determinants of gait speed. On average, women had a 0.054 m/s (95%CI:0.076-0.033, adjusted for height and age) lower mean gait speed compared to men. Higher BMI and lower physical activity were more strongly associated with lower gait speed in women compared to men (i.e. higher sensitivity). Lower educational level, living alone and having more chronic diseases, pain and depressive symptoms among women compared to men also contributed to observed lower gait speed in women (i.e. higher exposure). In conclusion, both a higher sensitivity and higher exposure to determinants of gait speed among women compared to men contributes to the observed lower gait speed among women aged 55 years and older. The identified (modifiable) contributing factors should be taking into account when developing prevention and/or treatment strategies aimed to enhance healthy physical aging. This might require a sex-specific approach in both research and clinical practice.

